# *Parvitermes* (Isoptera, Termitidae, Nasutitermitinae) in Central America: Two new termite species and reassignment of *Nasutitermes mexicanus*

**DOI:** 10.3897/zookeys.617.10040

**Published:** 2016-09-15

**Authors:** Rudolf H. Scheffrahn

**Affiliations:** 1 Fort Lauderdale Research and Education Center, University of Florida, Institute of Food and Agricultural Sciences, 3205 College Avenue, Davie, Florida, 33314, USA

**Keywords:** Neotropics, soldier key, enteric valve armature, new combination, taxonomy

## Abstract

The termite genus *Parvitermes* is now recognized on the Central American mainland to include *Parvitermes mexicanus*, **new combination** (previously in *Nasutitermes*) and two new species, *Parvitermes mesoamericanus* **sp. n.** and *Parvitermes yucatanus* **sp. n.**, herein described from soldiers and workers. These three species, nine West Indian *Parvitermes*, and *Antillitermes subtilis* all share characteristic enteric valve spines that orientate against intestinal flow. All species are subterranean nesters and cellulose feeders. Evidence is mounting that generic-level endemicity may be completely absent among the West Indian nasutitermitine fauna and that its origins stem from Central America.

## Introduction

Emerson (1949) erected the genus *Parvitermes* to accommodate six small nasutiform termites: *Nasutitermes brooksi* Snyder, 1925 from Cuba, *Constrictotermes discolor* Banks, 1919 and *Nasutitermes wolcotti* Snyder, 1924a from Puerto Rico, *Constrictotermes flaveolus* Banks, 1919 and *Constrictotermes pallidiceps* Banks, 1919 from Hispaniola, and *Nasutitermes laticephalus* Snyder, 1926 from Bolivia. Three additional *Parvitermes* species from Hispaniola were later added (*Parvitermes subtilis* Scheffrahn & Krecek, 1993, *Parvitermes collinsae* Scheffrahn & Roisin, 1995, ﻿and *Parvitermes dominicanae* Scheffrahn et al., 1998). The distribution of *Parvitermes brooksi* and *Parvitermes wolcotti* was expanded to include the central Bahamas (Scheffrahn et al. 2006) and the British and U.S. Virgin Islands (Scheffrahn et al. 2003), respectively.

Roisin et al. (1996) revised the small nasutes of the West Indies based mainly on worker morphology. The following taxa were reassigned to *Parvitermes*: *Constrictotermes toussainti* Banks, 1919, *Nasutitermes aequalis* Snyder, 1924b, and *Eutermes antillarum* Holmgren, 1910. Furthermore, *Parvitermes discolor* was placed in a new genus, *Caribitermes* Roisin, 1996, and *Parvitermes subtilis* was placed in another new genus, *Antillitermes* Roisin, 1996. The removal of *Parvitermes* as a Neotropical mainland genus was completed by Roisin et al. (1996), who transferred *Parvitermes laticephalus* to *Velocitermes*, and by Cuezzo and Cancello (2009), who showed that the Brazilian *Parvitermes bacchanalis* Mathews, 1977 should also be excluded from *Parvitermes*.

In the present paper, *Parvitermes* is shown to be a widespread endemic genus of the Central American mainland as *Parvitermes mexicanus* (Light, 1933), comb. n. and as two new Central American species, *Parvitermes mesoamericanus* and *Parvitermes yucatanus*. All three species are described mainly by the shape of soldier nasus and their enteric valve armature.

## Materials and methods

**Figure 1.**
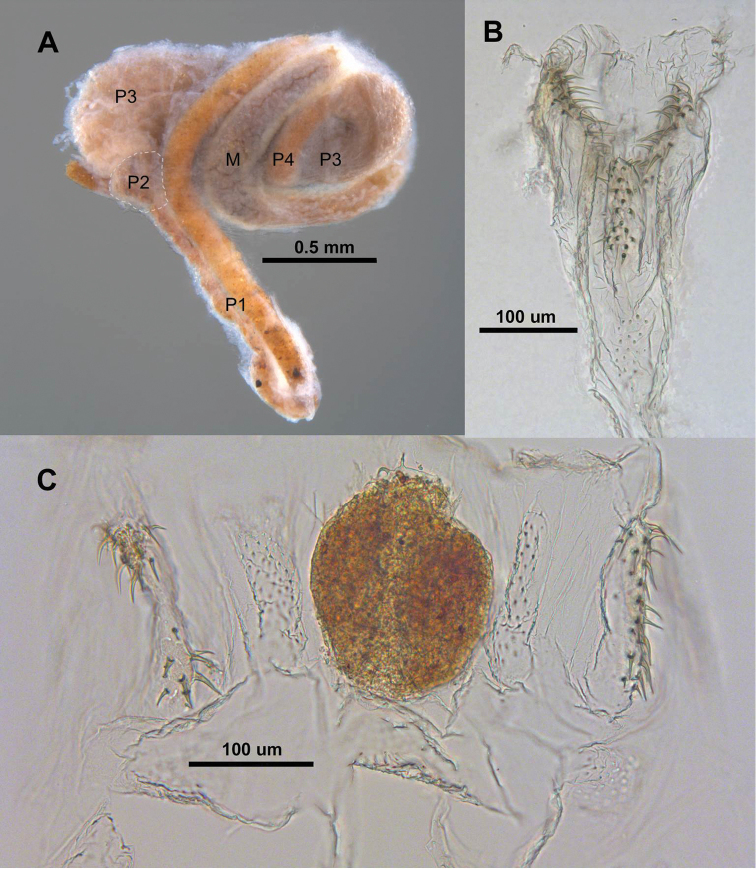
*Parvitermes brooksi*. **A** Dorsal left view of gut: M = mesenteron, P1-P4 = proctodeal segments 1-4 (limits of P2 highlighted) **B** Whole mount of P2 with musculature removed. Posterior (end attached to P3) at top of image **C** P2 splayed open; bacterial pellet attached to spines of the central pad.

**Figure 2.**
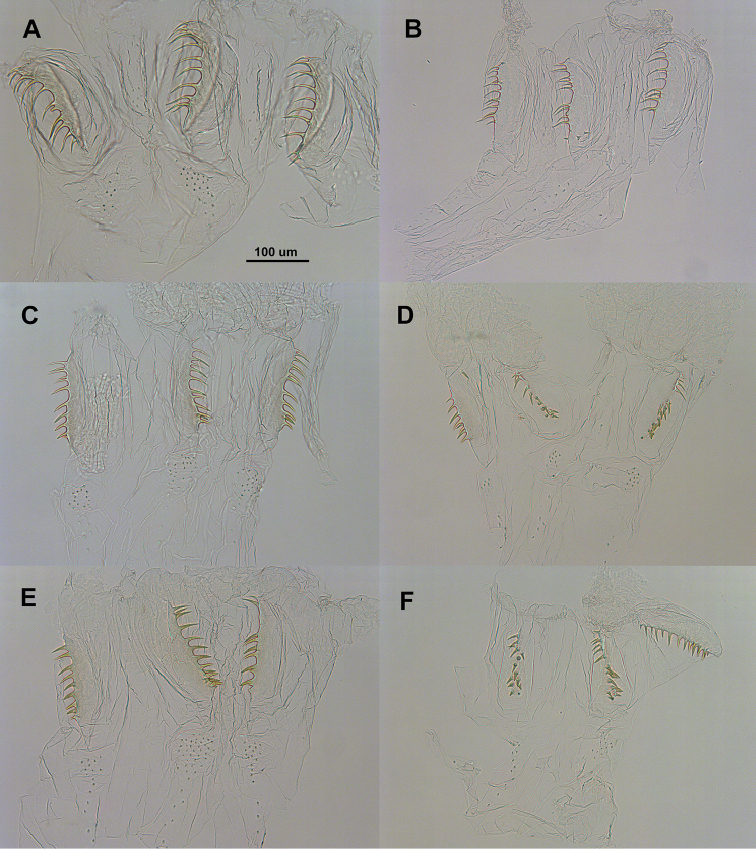
Enteric valve armatures. *Parvitermes mexicanus* comb. n., **A** worker **B** soldier. *Parvitermes mesoamericanus* sp. n. **C** worker **D** soldier. *Parvitermes yucatanus* sp. n. **E** worker **F** soldier. Posterior (end attached to P3) at top of each image.

**Figure 3.**
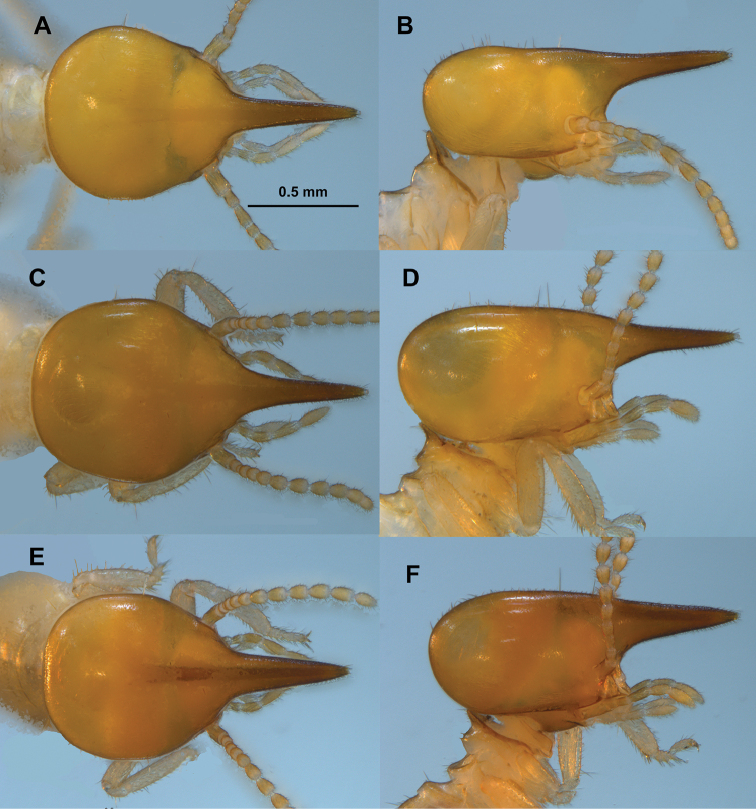
*Parvitermes* soldier head capsule. *Parvitermes mexicanus* comb. n., **A** dorsal **B** lateral. *Parvitermes mesoamericanus* sp. n. **C** dorsal **D** lateral. *Parvitermes yucatanus* sp. n. **E** dorsal **F** lateral.

**Figure 4.**
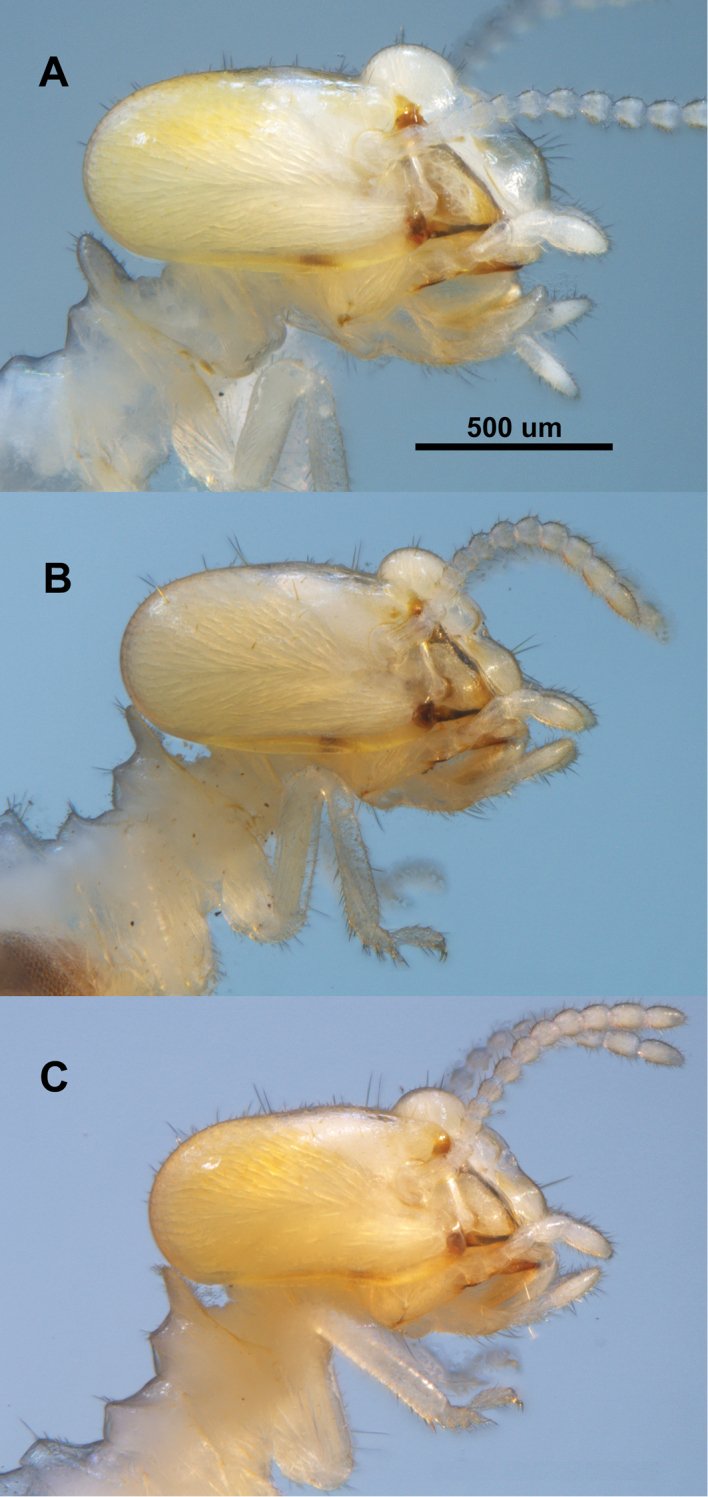
*Parvitermes* worker head and thorax, lateral views. **A** *Parvitermes mexicanus* comb. n. **B** *Parvitermes mesoamericanus* sp. n. **C** *Parvitermes yucatanus* sp. n.

**Figure 5.**
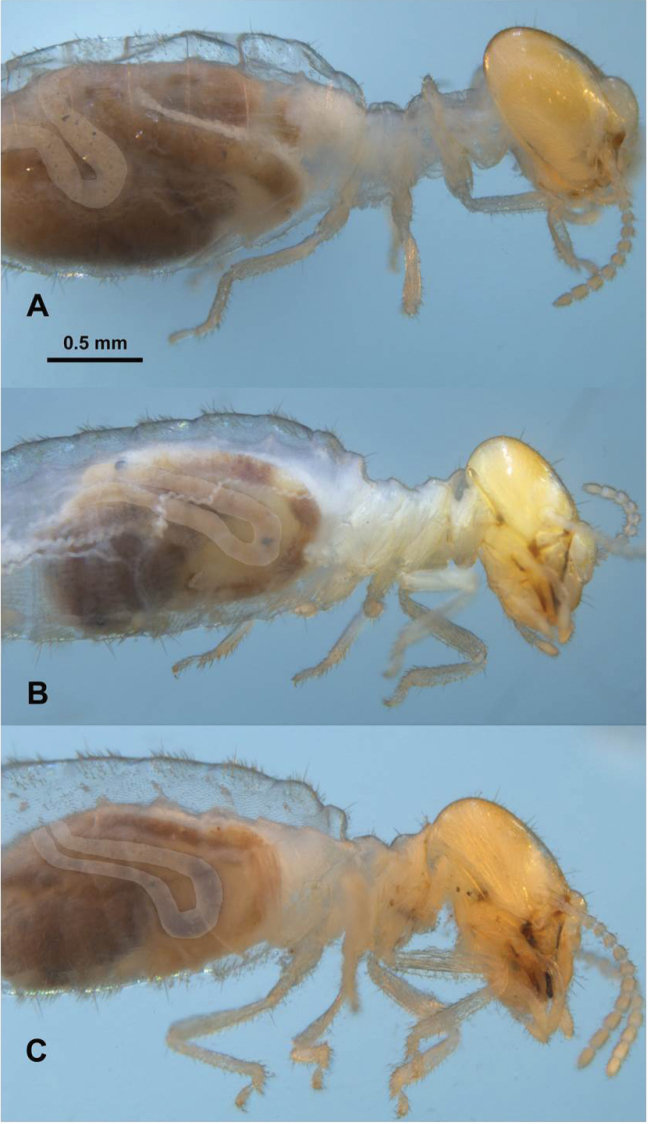
*Parvitermes* worker bodies with P1 highlighted. **A** *Parvitermes mexicanus* comb. n., dorsolateral view **B** *Parvitermes mesoamericanus* sp. n., ventrolateral view **C** *Parvitermes yucatanus* sp. n., ventrolateral view.

**Figure 6.**
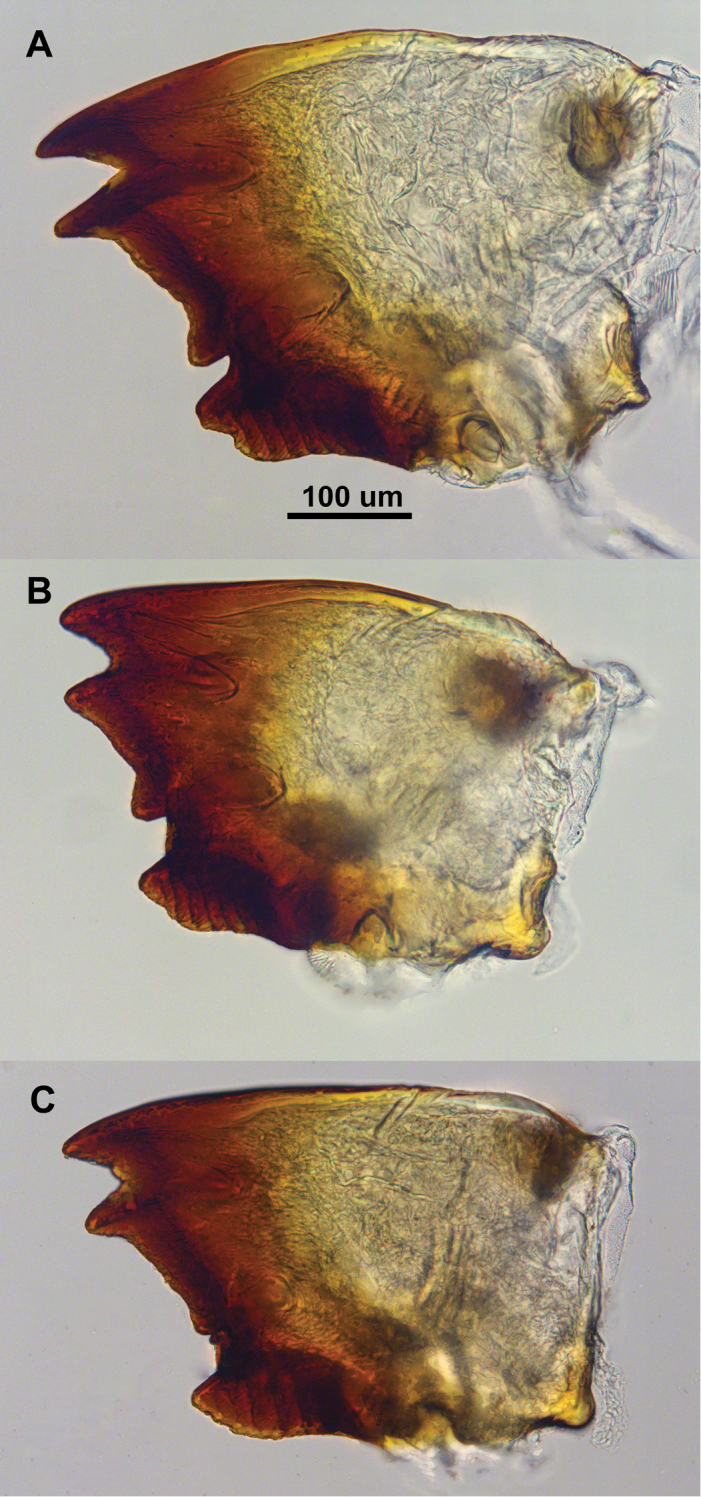
*Parvitermes* ventral views of left worker mandibles. **A** *Parvitermes mexicanus* comb. n. **B** *Parvitermes mesoamericanus* sp. n. **C** *Parvitermes yucatanus* sp. n.

**Figure 7.**
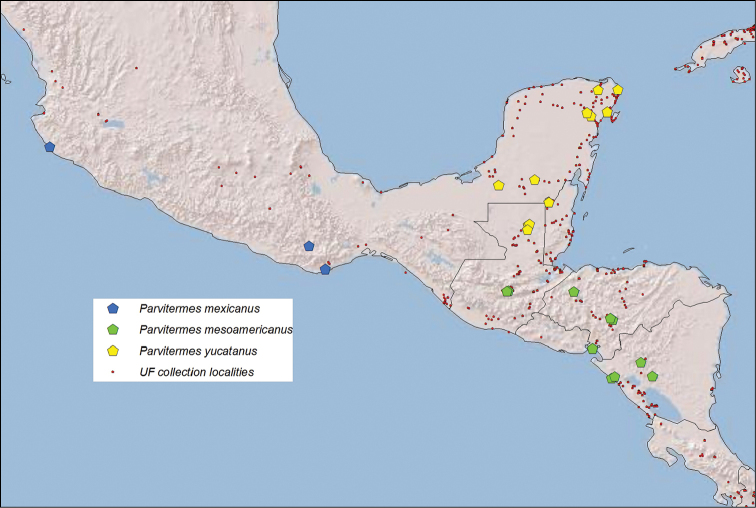
Collection localities of three *Parvitermes* species in the UF Termite collection. The far western sample of *Parvitermes mexicanus* comb. n. was taken near the type locality.

All material is from the University of Florida Termite Collection (UF) at the author’s address. Photographs (Figs 1A, 3–5) were taken as multi-layer montages using a Leica M205C stereomicroscope controlled by Leica Application Suite version 3 software. Preserved specimens were taken from 85% ethanol and suspended in a pool of Purell® Hand Sanitizer to position the specimens over a transparent Petri dish background. Microphotographs (Figs 1B, 1C, 2, 6) were taken from slide mounts in PVA medium (BioQuip, Rancho Dominquez, CA) using a Leica CTR 5500 compound microscope with bright field lighting. The distribution map (Fig. 7) was created using ArcGIS Desktop ver. 10.3 (ESRI, Redlands, CA).

## Taxonomy

### Key to soldiers of Central American species of *Parvitermes*

**Table:** 

1	Head capsule widest near posterior third (Fig. 3A), nasus angled slightly above plane of vertex (Fig. 3B)	***Parvitermes mexicanus***
–	Head capsule widest near middle (Fig. 3C, E), nasus angled in line or below plane of vertex (Fig. 3D, F)	**2**
2(1)	Nasus, in lateral view, nearly cylindrical in apical 2/3 (Fig. 3D)	***Parvitermes mesoamericanus* sp. n.**
–	Nasus, in lateral view, conical (Fig. 3F)	***Parvitermes yucatanus* sp. n.**

#### Genus Parvitermes

Taxon classificationAnimaliaIsopteraTermitidae

Emerson, 1949

##### Type species.

*Nasutitermes brooksi* Snyder, 1925. Type: soldier; Cuba, Cienfuegos, Soledad.

##### Remarks.

The nomenclatural summary for *Parvitermes* is provided by Krishna et al. (2013). The generic redescription of *Parvitermes* by Roisin et al. (1996) is relevant to all three *Parvitermes* species described herein with the exception that *Parvitermes mexicanus* comb. n. has a shorter first proctodeal segment (P1) compared to all others.

##### Diagnosis.

The spine arrangement and counter-current orientation of the *Parvitermes*
enteric valve armature (EVA), with the exception of *Antillitermes*, is unique among all termite genera. In addition to *Parvitermes*, only three other nasutitermitine genera are found from Mexico to Nicaragua, including *Nasutitermes*, *Subulitermes*, and *Tenuirostritermes* (*Atlantitermes* from Nicaragua in Scheffrahn et al. (2005) is an error). Compared to mainland *Parvitermes*, head capsules of *Nasutitermes* soldiers are larger and darker (with the exception of *Nasutitermes glabritergus* Snyder & Emerson, 1949 from Honduras, unpubl. record), those of *Subulitermes* are much smaller with much narrower cylindrical nasi, and the head capsules of *Tenuirostritermes* are very constricted near their middle.

##### Workers and soldiers.

The EVA arises within the second proctodeal segment (P2) which forms a swelling at the terminus of a very long (shorter and thicker in *Parvitermes mexicanus*), U-shaped P1. The P2 constricts somewhat at its attachment near the dorsal surface of the third proctodeal segment (P3 or paunch) to form a pear-shaped segment (Fig. 1A). The posterior EV ring (*sensu* Noirot 2001) of both workers and soldiers is uniquely composed of three keel-shaped pads covered with about 7-15 long spines directed into the P2 lumen (Fig. 1B). The spines are curved or angled counter to the direction of the food flow. The spiny pads are separated with or without additional patches of tiny conical teeth (Figs 1C, 2). In preserved specimens, the *Parvitermes* spines of each pad are imbedded into a congealed pellet of presumed bacterial cells (Fig. 1C).

#### Parvitermes mexicanus

Taxon classificationAnimaliaIsopteraTermitidae

(Light, 1933) comb. n.

Nasutitermes mexicanus Nickle & Collins, 1988 (soldier). Type localities: MEXICO: Colima: Colima, Jala, and Madrid.

##### Material examined.

MEXICO, 76 km S. Oaxaca, 16.49, -96.74, 11 Jan 1997, T.G. Myles & D.A. Muruvanda, UF no. MX23; Chamela, 19.5314, -105.0832, 1 Apr 1996, G. Thompson, MX99; Aguaje de la Anona, 15.7731, -96.2168 , 27 May 2006, T. Atkinson, MX572. Soldiers of these three colony samples were identified based on the following: congruence with both dorsal and lateral head capsule photographs from the original description (Figs L, O, Light 1933), Light’s 1933 rostrum (nasus) diagnosis stating that it is “slightly uplifted distally”, proximity of the Chamela sample to the type localities, and Light’s 1933 measurements. Furthermore, scanning electron micrographs of a *Parvitermes mexicanus* soldier from Chamela (figs 15D, H, Nickle and Collins 1988) agree perfectly with the examined soldiers.

##### Comparisons.

See below under *Parvitermes mesoamericanus* sp. n.

##### Alate.

Unknown.

##### Soldier

(Table 1, Fig. 2B, 3A, B). Monomorphic; however some rare divergent size morphs reported by Light 1933. Head capsule and pronotum light orange-brown; nasus darker. In dorsal view, cephalic gland duct partially or completely visible from nasus to reservoir. Many small and a few longer setae scattered on head; setae on nasus very small and numerous.

**Table 1.** Measurements (mm) of *Parvitermes mexicanus* comb. n. soldiers.

Colony	Head length to end of nasus	Head width (max.)	Pronotal width	Hind tibia length
MX23 (n=3)	1.44–1.52	0.80–0.82	0.42–0.46	0.94–0.96
MX99 (n=2)	1.50–1.52	0.88–0.92	0.44–0.45	0.92–0.93
MX572 (n=8)	1.34–1.46	0.72–0.80	0.44–0.46	0.89–0.96
Range	1.34–1.52	0.72–0.92	0.42–0.46	0.89–0.96
Mean	1.43	0.80	0.45	0.92

In dorsal view, head capsule outline, without nasus, subtrapazoidal; nasus about 2/3 as long as rest of head capsule; head capsule slightly constricted behind antennal sockets; widest at posterior 1/3. In lateral view, vertex with slight concavities near midpoint; second slight concavity at base of nasus; plane of vertex parallel with ventral margin of head capsule. In dorsal view nasus is narrowly conical, about twice its width at base compared to midpoint. In lateral view nasus narrowly conical; angled ca. 5°above plane of vertex. Mandibles without points. Antennal with 13 articles (1>2<3>4). Hind tibia longer than head width. Pronotum with scattered microscopic setae (0.03 mm); anterior lobe evenly convex and ca. 90° from plane of posterior lobe, posterior lobe more blunt. Each tergite with 3-4 long (0.1 mm) setae and dozens of microscopic (0.03 mm) setae. EVA consists of three irregular rows of sharp, narrow, and down-curved spines; a few small scale-like spines in the anterior ring.

##### Worker

(Table 2, Figs 2A, 4A, 5A, 6A). Monomorphic. Head capsule very pale yellow with two slightly darker yellowish-orange dorso-lateral patches; pronotum very pale yellow; body, antennae, and legs hyaline. Antennal with 12 articles. Postclypeus considerably inflated in lateral view; scattered short and medium setae on head capsule. Abdomen with many short and a few scattered longer setae. Mandibles with about eight ridges on molar plate, molar plate with distinct dorsal notch; apical and first marginal teeth of similar shape and projection; third marginal smaller, separated from first by slightly concave cutting edge. Gut with P1 U-shaped turn near midpoint, bottom of turn extending only to dorso-lateral aspect of abdomen. EVA consists of three irregular rows of about 10-12 sharp, narrow, and down-curved spines; anterior ring with three patches of small scale-like spines.

**Table 2.** Measurements (mm) of *Parvitermes mexicanus* comb. n. workers.

Colony	Head length to end of postclypeus	Postclypeal length	Head width	Pronotal width	Hind tibia length
MX23 (n=3)	0.84–1.06	0.21–0.22	0.88–1.06	0.44–0.48	0.72–0.84
MX99 (n=1)	0.92	0.23	0.96	0.42	0.74
MX572 (n=11)	0.80–1.01	0.21–0.26	0.90–1.04	0.40–0.55	0.74–0.94
Range	0.80–1.06	0.21–0.26	0.88–1.06	0.40–0.55	0.72–0.94
Mean	0.93	0.23	0.96	0.44	0.78

##### Distribution.

Tropical Pacific slope of Mexico (Fig. 7).

#### Parvitermes mesoamericanus sp. n.

Taxon classificationAnimaliaIsopteraTermitidae

http://zoobank.org/C83A167F-3E4F-43DE-9329-9232DD812A92

##### Type-locality.

Honduras, S. Pinalillo, 15.0860, -88.2160, 144 m elev.

##### Holotype.

Soldier, 2 Jun 2007, Scheffrahn et al. cols., UF no. HN822 (in microvial).

##### Paratypes.

GUATEMALA: Salama, 15.1055, -90.3261 , 28 May 2006, Scheffrahn et al., GUA16; Road to Rabinal, 15.1045, -90.3722, 28 May 2006, Scheffrahn et al., GUA33; HONDURAS: Coyolito, 13.3149, -87.6227, 31 May 2007, Scheffrahn et al., HN431; NICARAGUA: Los Cardones, 12.8851, -86.0534, 30 May 2004, Scheffrahn et al., NI114.

##### Imago.

Unknown.

##### Soldier

(Table 3, Figs 2D, 3CD, 8). Monomorphic. Head capsule and pronotum light orange-brown; nasus darker. In dorsal view, cephalic gland duct partially or completely visible from nasus to reservoir. Many small, some medium, and a few longer setae scattered on head; setae on nasus small and numerous. In dorsal view, head capsule outline, without nasus, ovoid; nasus about 2/3 as long as rest of head capsule; head capsule barely constricted behind antennal sockets; widest in middle. In lateral view, vertex nearly in a flat plane; vertex and ventral margin of head capsule converge to front. In dorsal view nasus is narrowly conical, about thrice its width at base compared to midpoint; In lateral view nasus conical; projecting directly anterior below plane of vertex. Mandibles with short, very narrow, points. Antennal with 12 articles (1>2<3=4). Hind tibia about as long as or shorter than maximum head width. Pronotum with scattered microscopic setae (0.05 mm); anterior lobe evenly convex and ca. 90° from plane of posterior lobe, posterior lobe more blunt. Each tergite with 3-4 long (0.13 mm) setae and dozens of microscopic (0.05 mm) setae. EVA consists of three irregular rows of about 8-12 long subtriangulate, and very slightly down-curved spines; a few small scale-like spines in the anterior ring.

**Table 3.** Measurements (mm) of *Parvitermes mesoamericanus* sp. n. soldiers.

Colony	Head length to end of nasus	Head width (max.)	Pronotal width	Hind tibia length
GUA16 (n=12)	1.38–1.50	0.76–0.84	0.36–0.41	0.66–0.78
GUA33 (n=12)	1.48–1.63	0.83–0.91	0.42–0.48	0.76–0.84
HN431 (n=12)	1.37–1.49	0.76–0.82	0.41–0.43	0.68–0.76
HN822 (n=10)	1.37–1.46	0.71–0.80	0.36–0.39	0.64–0.75
NI114 (n=12)	1.30–1.43	0.69–0.75	0.40–0.42	0.64–0.75
Range	1.30–1.63	0.69–0.91	0.36–0.48	0.64–0.84
Mean	1.44	0.78	0.40	0.72

**Figure 8.**
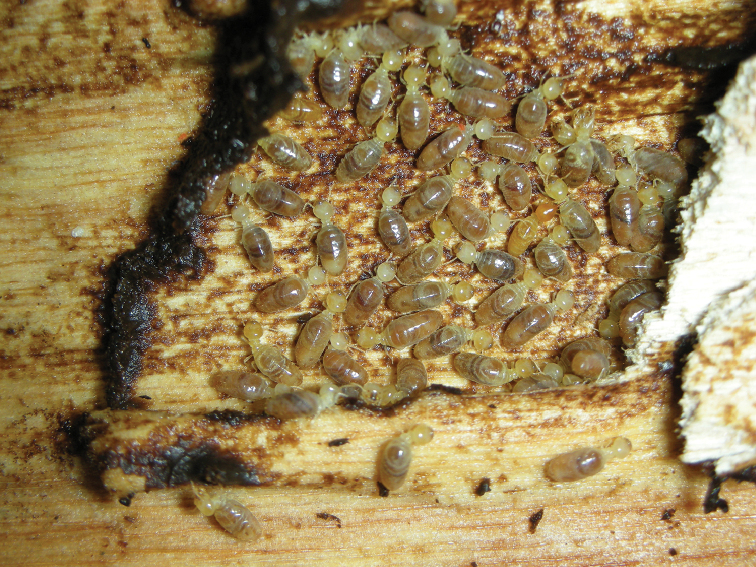
Field photograph of *Parvitermes mesoamericanus* sp. n. foragers feeding within a crevice of damp wood (Coyolito, Honduras; paratype locality, HN431).

##### Worker

(Table 4, Figs 2C, 4B, 5B, 6B, 8). Monomorphic. Head capsule very pale yellow with two slightly darker yellowish-orange dorso-lateral patches; pronotum very pale yellow; body, antennae, and legs hyaline. Antennal with 12-13 articles. Postclypeus considerably inflated in lateral view; scattered short, medium, and a few longer setae on head capsule. Abdomen with many short and longer setae. Mandibles with about eight ridges on molar plate, molar plate with slight dorsal notch; apical and first marginal teeth of similar shape and projection; third marginal smaller, separated from first by slightly emarginate cutting edge. Gut with very long P1; U-shaped turn near midpoint, bottom of turn extending to ventro-lateral aspect of abdomen. EVA consists of three irregular rows of about 8-12 long subtriangulate, and very slightly down-curved spines; three patches with small scale-like spines in the anterior ring.

**Table 4.** Measurements (mm) of *Parvitermes mesoamericanus* sp. n. workers.

Colony	Head length to end of postclypeus	Postclypeal length	Head width	Pronotal width	Hind tibia length
GUA16 (n=12)	0.74–0.92	0.17–0.23	0.80–0.92	0.39–0.52	0.54–0.75
GUA33 (n=12)	0.85–0.98	0.20–0.23	0.87–0.92	0.44–0.54	0.63–0.80
HN431 (n=12)	0.70–0.88	0.17–0.20	0.77–0.90	0.37–0.48	0.53–0.70
HN822 (n=10)	0.76–0.86	0.17–0.20	0.76–0.86	0.36–0.46	0.58–0.74
NI114 (n=12)	0.76–0.85	0.18–0.20	0.77–0.86	0.40–0.46	0.60–0.75
Range	0.70–0.98	0.17–0.23	0.76–0.92	0.36–0.54	0.53–0.80
Mean	0.83	0.20	0.84	0.45	0.66

##### Etymology and distribution.

Named for Middle America which encompasses Guatemala, Honduras, and Nicaragua; the known range of this termite. The distribution habitat of *Parvitermes mesoamericanus* (Fig. 7) is more xeric than adjacent regions lacking this termite.

##### Comparisons.

The soldier of *Parvitermes mesoamericanus* has the nasus directed forward, the head capsule widest in the middle, a few scattered long setae on the vertex, and points on the mandibular stubs while in *Parvitermes mexicanus*, the nasus is slightly upturned, the head is widest in the posterior third, the vertex lacks scattered long setae, and the mandibular stubs have points. The worker of *Parvitermes mesoamericanus* has a much longer and more ventrally positioned P1, stouter and less curved EVA spines, and longer setae on the vertex, while in *Parvitermes mexicanus* the P1 is shorter and more dorsal, the EVA spines are thinner and more curved, and the setae on the vertex are shorter. The *Parvitermes mesoamericanus* worker is proportionally smaller to its soldier as compared *Parvitermes mexicanus*. Both castes of *Parvitermes mexicanus* have longer hind tibia than *Parvitermes mesoamericanus*.

#### Parvitermes yucatanus sp. n.

Taxon classificationAnimaliaIsopteraTermitidae

http://zoobank.org/0A5B0FFA-48A8-4DF0-84EA-0051AD9CA0E5

##### Type-locality.

**Mexico**, 0.9 km N. gate of Punta Sam, 21.2423, -86.8056, 2 m elev.

##### Holotype.

Soldier. 9 Dec 1997, J. Chase, J. Mangold cols., UF col. no. MX161 (in microvial).

##### Paratypes.

GUATEMALA: P. N. Tikal, 17.1371, -89.6803, 30 May 2006, Scheffrahn et al., GUA222; MEXICO: Hwy 307, 1 km S Marine, 20.5803, -87.1424, 8 Dec 1997, J. Chase, J. Mangold, MX148; same data, MX152; Chicana Ecovillage, 18.5178, -89.4846, 21 Jan 2001, MX281; 10.5 km W Coba toward Chemax, 20.5514, -87.8049, 22 Jan 2003, J. Chase, J. Mangold, MX492.

##### Alate.

Unknown.

##### Soldier.

(Table 5, Figs 2F, 3E, F). In all respects, similar to *Parvitermes mesoamericanus* except for the following: In dorsal view nasus conical, about 1.6x its width at base compared to midpoint; in lateral view nasus broadly conical. Mandibles with short, very narrow, points. Antennal with 11-12 articles (1>2<3=4). Hind tibia usually shorter than maximum head width. Pronotum with a few longer setae (0.10 mm) along margin of anterior lobe.

**Table 5.** Measurements (mm) of *Parvitermes yucatanus* sp. n. soldiers.

Colony	Head length to end of nasus	Head width (max.)	Pronotal width	Hind tibia length
GUA222 (n=12)	1.36–1.45	0.70–0.78	0.36–0.40	0.65–0.70
MX148 (n=2)	1.28–1.29	0.66	0.36	0.58
MX152 (n=12)	1.34–1.42	0.68–0.74	0.34–0.40	0.64–0.70
MX161 (n=12)	1.36–1.46	0.74–0.78	0.38–0.44	0.66–0.74
MX281 (n=12)	1.38–1.45	0.72–0.78	0.34–0.39	0.64–0.76
MX492 (n=12)	1.32–1.42	0.72–0.78	0.34–0.38	0.64–0.70
Range	1.28–1.46	0.66–0.78	0.34–0.44	0.58–0.76
Mean	1.38	0.72	0.37	0.67

##### Worker.

(Table 6, Figs 2E, 4C, 5C, 6C). In all respects, similar to *Parvitermes mesoamericanus* except for the following: Mandibles with about seven ridges on molar plate, molar plate without dorsal notch; apical and first marginal teeth of similar shape and projection; third marginal smaller, separated from first by slightly concave cutting edge. EVA consists of three irregular rows of about 7–12 long, narrow, subtriangulate, and slightly down-curved spines.

**Table 6.** Measurements (mm) of *Parvitermes yucatanus* sp. n. workers.

Colony	Head length to end of postclypeus	Postclypeal length	Head width	Pronotal width	Hind tibia length
GUA222 (n=12)	0.73–0.85	0.17–0.20	0.76–0.86	0.36–0.46	0.54–0.67
MX148 (n=4)	0.73–0.80	0.17–0.19	0.73–0.80	0.36–0.39	0.51–0.58
MX152 (n=12)	0.68–0.84	0.18–0.23	0.71–0.85	0.32–0.44	0.54–0.70
MX161 (n=12)	0.78–0.87	0.17–0.19	0.77–0.84	0.40–0.53	0.56–0.74
MX281 (n=12)	0.76–0.84	0.17–0.19	0.78–0.84	0.36–0.44	0.60–0.74
MX492 (n=12)	0.75–0.85	0.17–0.19	0.74–0.82	0.36–0.40	0.54–0.67
Range	0.68–0.87	0.17–0.23	0.71–0.86	0.32–0.53	0.51–0.74
Mean	0.78	0.18	0.79	0.38	0.61

##### Etymology and distribution.

Named for the Yucatan Peninsula which encompasses Belize, Mexico, and Guatemala; the known range of *Parvitermes yucatanus* (Fig. 7). This region has a pronounced dry winter season.

##### Comparisons.

The soldiers of *Parvitermes yucatanus* and *Parvitermes mesoamericanus* are very similar with the following exception: the nasus of *Parvitermes yucatanus*, in lateral view, is more conical and broader at the base than that of *Parvitermes mesoamericanus*. The workers of *Parvitermes yucatanus* and *Parvitermes mesoamericanus* are indistinguishable. The distributions of *Parvitermes yucatanus* and *Parvitermes mesoamericanus* appear to be allopatric (Fig. 7) with the latter species occupying a more arid zone.

##### Biology.

The Central American *Parvitermes* are wood-surface feeders. They typically attack wood in contact with the ground where they encase their surroundings with dark carton material (Fig. 8) reminiscent of *Amitermes* and build narrow foraging galleries to above-ground feeding sites (Light 1933, Weesner 1970 for *Parvitermes mexicanus*). Colonies nest in the soil underneath rocks and logs where brood and larvae have been found in weak cells of thin dark carton. In the West Indies, *Parvitermes* are often collected in hollowed-out stems of woody herbaceous plants (*Parvitermes brooksi* and *Parvitermes wolcotti*). In the arid lands of the Dominican Republic, *Parvitermes flaveolus* attacks wooden fence posts, and after rains, will feed on dried grass bunches that they cover with a thin arcade of soil.

## Discussion

The current study reveals that *Parvitermes* is no longer a genus exclusive to the West Indies (Roisin et al. 1996) but has a widespread mainland complement of three species. This leaves only the monospecific genera *Antillitermes* and *Caribitermes* as the remaining endemics of all termite genera in the West Indies (excluding the continental islands of Trinidad and Tobago). The gestalt of the *Antillitermes subtilis* EVA closely resembles that of *Parvitermes* s. str. (Roisin et al. 1996) and suggests that the EVA is a mainland synapomorphy of both genera. *Antillitermes subtilis* may very well be a species of *Parvitermes*. *Caribitermes discolor* may also have a mainland lineage as it resembles an undescribed species from Panama (PN1315, Scheffrahn unpubl.). Therefore, it is quite possible, with the exception of the relict *Constrictotermes guantanamensis* from Cuba (Krěcěk et al. 1996), that all West Indian termites share congeneric species on the Central American mainland and that the West Indian fauna arose from Pleistocene/Miocene (Krishna and Grimaldi 2009) overwater dispersal events from Central America (Darlington 1938, Hedges 1996) or the more recent late Pleistocene land connections (Scheffrahn et al. 2006).

## Supplementary Material

XML Treatment for Parvitermes

XML Treatment for Parvitermes mexicanus

XML Treatment for Parvitermes mesoamericanus

XML Treatment for Parvitermes yucatanus
